# The hydrogen storage nanomaterial MgH_2_ improves irradiation-induced male fertility impairment by suppressing oxidative stress

**DOI:** 10.1186/s40824-022-00266-6

**Published:** 2022-05-26

**Authors:** Jing Ma, Suhe Dong, Hongtao Lu, Zhongmin Chen, Huijie Yu, Xuejun Sun, Renjun Peng, Wei Li, Sinian Wang, Qisheng Jiang, Fengsheng Li, Li Ma

**Affiliations:** 1 The Postgraduate Training Base of Jinzhou Medical University (The PLA Rocket Force Characteristic Medical Center), Beijing, 100088 China; 2 PLA Rocket Force Characteristic Medical Center, Beijing, 100088 China; 3 Department of Naval Medicine, Naval Medical University, Shanghai, 200433 China; 4 Center of Hydrogen Science, Shanghai Jiao Tong University, Shanghai, 200030 China

**Keywords:** Nanomaterial, Irradiation damage, Male infertility, ROS, Hydroxyl radical

## Abstract

**Objective:**

This study aimed to reveal the protective effect of hydrogen storage nanomaterial MgH_2_ on radiation-induced male fertility impairment.

**Methods:**

The characterization of MgH_2_ were analyzed by scanning electron microscopy (SEM) and particle size analyzer. The safety of MgH_2_ were evaluated in vivo and in vitro. The radioprotective effect of MgH_2_ on the reproductive system were analyzed in mice, including sperm quality, genetic effect, spermatogenesis, and hormone secretion. ESR, flow cytometry and western blotting assay were used to reveal the underlying mechanisms.

**Results:**

MgH_2_ had an irregular spherical morphology and a particle size of approximately 463.2 nm, and the content of Mg reached 71.46%. MgH_2_ was safe and nontoxic in mice and cells. After irradiation, MgH_2_ treatment significantly protected testicular structure, increased sperm density, improved sperm motility, reduced deformity rates, and reduced the genetic toxicity. Particularly, the sperm motility were consistent with those in MH mice and human semen samples. Furthermore, MgH_2_ treatment could maintain hormone secretion and testicular spermatogenesis, especially the generation of Sertoli cells, spermatogonia and round sperm cells. In vitro, MgH_2_ eliminated the [·OH], suppressed the irradiation-induced increase in ROS production, and effectively alleviated the increase in MDA contents. Moreover, MgH_2_ significantly ameliorated apoptosis in testes and cells and reversed the G2/M phase cell cycle arrest induced by irradiation. In addition, MgH_2_ inhibited the activation of radiation-induced inflammation and pyroptosis.

**Conclusion:**

MgH_2_ improved irradiation-induced male fertility impairment by eliminating hydroxyl free radicals.

**Graphical abstract:**

Mice fertility and function were evaluated with or without MgH_2_ treatment after 5 Gy irradiation. MgH_2_ had the ability of hydroxyl radicals scavenging and MDA suppressing in testicular tissue induced by irradiation. Further, MgH_2_ could participate in spermatogenesis and protect sperm development in three stages: the generation of Sertoli cells (*Sox-9+*), spermatogonia (*Stra8+*) and round sperm cells (*Crem+*). Moreover, MgH_2_ alleviated the decrease of testosterone secreted by interstitial cells after irradiation. In addition, MgH_2_ suppressed apoptosis, pyroptosis and inflammatory response and alleviated cell cycle arrest by mediating IR-induced ROS.
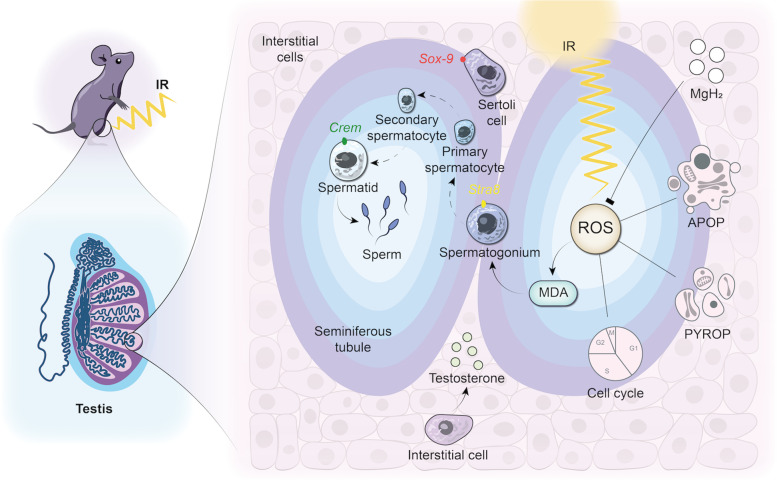

**Supplementary Information:**

The online version contains supplementary material available at 10.1186/s40824-022-00266-6.

## Introduction

Total body irradiation due to accidental exposure or overexposure and abdominal irradiation during radiotherapy for bladder cancer or prostatic cancer can result in the accumulation of harmful irradiation in the testes [1, 2]. The testes are very sensitive to irradiation. Irradiation doses as low as 0.5 Gy can lead to infertility and spermatogenic dysfunction [3, 4]. In a follow-up survey of 666 bladder cancer patients who had undergone pelvic radiotherapy, 40% of men exhibited subnormal testosterone (T) levels, and T levels lower than 10 nM can serve as a marker of hypogonadism [3]. In an analysis of 18 patients treated with external prostatic irradiation, which is up to 3% of the prescribed dose for prostate cancer, a mean of 2.2 Gy (range, 1.2 Gy to 5.4 Gy) was measured in the testes [5]. At present, approximately 15% of couples of childbearing age suffer from infertility worldwide. In some developing countries, the infertility rate is even as high as approximately 30%. For more than 50% of couples, infertility is caused by decreased sperm quality and defects in sperm function, and irradiation exposure is an important risk factor for these defects [6]. In a retrospective study that analyzed the fertility of 67 cancer patients, 90% of the patients were treated with radiotherapy, and 57% of the patients were azoospermic after treatment [7]. Therefore, it is urgent to decrease the risks of male infertility associated with irradiation.

Radiation-induced damage to the male reproductive system mainly includes ultrastructural changes in the seminiferous epithelium of the testes, the epididymal epithelium, and the seminiferous tubule epithelium as well as dysfunction of testicular spermatogenesis [3]. Spermatogonia are uniquely sensitive to irradiation and can be completely destroyed by an irradiation dose of 3 Gy [8]. ROS in irradiated testicular tissue are mainly generated by hydrolytic dissociation, which can damage all biological molecules in mammals, including DNA, RNA, proteins and lipids [3]. In the male reproductive system, ROS lead to mutations in or apoptosis of spermatogenic cells, decreased sperm number and motility, and increased sperm deformity rate [9]. The generation of large amounts of ROS induces apoptosis via the mitochondrial pathway. Additionally, ROS can also cause cell cycle arrest in the G1/S and G2/M phases [10] and can activate and mediate the secretion of cytokines to trigger pyroptosis [11].

Most strategies to preserve male fertility are still in clinical trials and cannot fundamentally address this issue. Therefore, more effective strategies for treating fertility impairment are needed. Protection against radiation damage is mainly based on free radical scavenging. Free radical scavengers can alleviate IR-induced oxidative damage by inhibiting free radical formation, removing free radicals, enhancing DNA repair, reducing postirradiation inflammatory responses, and even delaying cell division to allow cells more time for self-repair [12]. Most free radical scavengers are still in preclinical research; only WR-2721 has been approved by the FDA for use in the clinic to protect salivary glands in head and neck cancer patients undergoing treatment [13], but it results in clear toxicity when administered clinically. Molecular hydrogen (H_2_) has been reported to selectively scavenge [·OH] and has the potential to become a radioprotective agent [14]. H_2_ inhibited IR-induced acute injury, reduced ROS levels and alleviated long-term fibrosis injury in mouse lungs [10]. Furthermore, treatment with H_2_ has been proven to significantly increase sperm number and promote spermatogenesis after testis irradiation [15]. However, the rich hydrogen aqueous solution has some disadvantages, including inconvenient storage and transportation, low hydrogen concentration, and insufficient antioxidant capacity. Therefore, it is necessary to develop more powerful free radical scavengers to protect male fertility during irradiation.

Recently, advanced nanomaterials have been applied in radiation biology [16]. Owing to their large specific surface area, micro- or nanomaterials accelerate biochemical reactions on their surfaces. Moreover, nanoparticles are light in weight, high in mass density, nontoxic and easy to store [17]. MgH_2_ nanomaterials, which are used for hydrogen storage and release, have the advantages of good stability, high hydrogen storage capacity and smooth hydrogen release capacity [18]. In this study, we explored the structural characteristics of MgH_2_ micro- and nanomaterials with excellent dispersion properties, confirmed the biosafety of MgH_2_ for both liver and renal function and cell viability, verified the potential radioprotective effect of MgH_2_ on male fertility, including the improvement in sperm motility and semen quality and the maintenance of spermatogenesis after ionizing radiation exposure, and revealed the mechanisms by which MgH_2_ suppresses the inflammation, apoptosis, pyroptosis and cell cycle arrest induced by irradiation through ROS.

## Materials and methods

### Characterization

The MgH_2_ particle size was evaluated using Nano-ZS90 dynamic light scattering (Malvern Instruments, Malvern, UK), it was measured after particle dispersion with corn oil. The morphology and elemental composition of MgH_2_ were analyzed by SEM (REGULUS-8230).

### Animals, cells and human samples

Male C57BL/6 mice, 8 weeks old, weighing 20 ± 2 g, were purchased from Speifu Biotechnology Co., Ltd. (Beijing, China). All the experimental procedures were carried out in accordance with the ARRIVE Guidelines and the U.K. Animals (Scientific Procedures) Act, 1986 and associated the National Research Council’s Guide for the Care and Use of Laboratory Animals.

Mouse GC-2 spd spermatocytes purchased from procell life Science&Technology Co.,ltd (Wuhan, China), which were cultured in DMEM supplemented with 10% fetal bovine serum (Gibco, USA), 100 U/mL penicillin and 100 mg/mL streptomycin (Gibco, USA). The cells were cultured in an incubator at 37 °C in 5% CO_2_.

We tested the effect of MgH_2_ treatment on sperm motility after irradiation in human semen samples, followed the principles of the code of ethics of the World Medical Association (Helsinki Declaration) for experiments involving humans and approved by the ethics committee of the PLA Rocket Force Characteristic Medical Center (KY2021037).

### Treatment

MgH_2_ is provided by Shanghai Jiao Tong University. MgH_2_ was briefly prepared as follows [19]: first, the magnesium-based material is uniformly heated under the protection of inert gas, and magnesium vapor is formed when the magnesium reaches its vaporization temperature. Then, the magnesium vapor is cooled to produce magnesium powder, and hydrogen is mixed into the reaction. Finally, under a certain temperature and pressure, the magnesium powder and hydrogen are heated again to fully react to form magnesium hydride (MgH_2_).

Mice were randomly divided into four groups: the Blank, MgH_2_, IR, and IR + MgH_2_ groups. The mice in the Blank and IR groups were administered deionized water, and the mice in the other groups were administered MgH_2_ at a concentration of 10 μg/ml in 0.1 ml per mouse for 3 days via the intragastric route. Irradiation was performed on the third day after administration, and MgH_2_ was administered immediately after irradiation. The mice in the IR and IR + MgH_2_ groups were exposed to irradiation (KUBTEC XCELL 225, 225 KV 13.2 mA, 1 Gy/min). MgH_2_-supplemented feed (0.5% in mass) was given to MH group mice; the corresponding control feed was given to MG group mice.

GC-2 spd cells were irradiated with a single dose of 10 Gy X-ray. Irradiation was carried out at room temperature, and unirradiated control cells were studied in parallel under the same conditions.

### ESR assay

The Feton reaction was performed to generate [·OH]. Ten microliters of 400 mmol/L DMPO, 0.4 mmol/L ferrous sulfate, deionized water (pH 4.5), and 4% H_2_O_2_ were quickly mixed. The ESR spectrum was recorded between 330 and 340 mT. Ten microliters of different concentrations of MgH_2_ were added to the Fenton reaction system, and the ESR spectrum was observed.

### Body weight and testicular index assay

The testicular weight and body weight of the same mice were measured and recorded, and these values were used to calculate the testicular index as follows: testicular index = testicular weight/body weight (mg/g).

### Sperm motility and count assay

Sperm suspensions were obtained according to the methods described in the literature [20]. The samples were analyzed with a sperm automatic analyzer (CASA, Zeiss Lab.Ai,GER). Then, the sperm counts were determined by counting under a microscope. The sperm suspensions were stained with eosin dye to observe sperm malformation. Abnormal spermatozoa were observed by optical microscopy (DMI8A, Leica, Germany) (200 X). A total of 200 sperms in each sample were observed.

### Genetic effect assay

Twenty-one days after irradiation, male mice were caged with normal female mice in ratios of two females and one male per cage. The pregnancy duration, the number of offspring per litter and the number of stillbirths per litter were recorded. The mice in the F0 and F1 generations were killed 6 hours after colchicine injection. The testicles were removed and placed in 0.4% potassium chloride at 37 °C. The fixing solution was added after 30 minutes. After 8 min, the samples were centrifuged at 1000 rpm/min for 8 min. This procedure was repeated once. The samples were adjusted to a volume of 0.5 ml, mixed thoroughly, dropped on a prechilled slide, allowed to dry, and then stained with Giemsa liquid. After washing with clear water, the samples were air dried, and the chromosome morphology was use Chromosome image analysis system observed under Metafer- Automated Slide Scanning Platform (zeiss, AXio Imager Z2, GER) (200 X).

### Pathological assay

On the 1st, 29th and 35th days after irradiation, the testis and epididymis were separated, fixed in 4% paraformaldehyde, fixed on slides and analyzed according to the methods H&E, immunofluorescence and immunohistochemical staining. Primary antibodies (*Ki67, stra8, Crem and Sox9*) and secondary antibodies were utilized to analyze the slides using an optical microscope. Slides were captured by a fluorescence microscope. The epithelial thickness (from basement membrane to lumen) and diameter of 30 seminiferous tubules in randomly selected mouse testes 35 days after IR were measured by Image-Pro Plus software. For *Tunel* staining, the slides were processed according to the protocol of the kit (Servicebio, Wuhan, China).

### ROS assay

The level of ROS in GC2 cells was measured using a kit according to the manufacturer’s instructions (A507, Gene Copoeia) and detected by flow cytometry (BD FACSCalibur, USA).

### Hormone and biochemical assay

The mice were sacrificed before blood collection. After coagulation, the blood samples were centrifuged at 4 °C and 3000 g for 15 minutes. The supernatants were analyzed to measure the FSH, LH, T, AST and ALT levels. The MDA levels were measured according to the instructions of a kit (S0131S, Beyotime, China).

### Western blotting assay

Mouse testis tissues or cells were lysed with RIPA lysis buffer. The protein concentrations were determined by a BCA protein detection kit (Beyotime, China). Western blotting was conducted following standard procedures. The antibodies used in this study were as follows: *Caspase-3, Cleaved caspase-3, Bax, Puma, Cytochrome-c, Parp, Cleaved Parp, p21, p27, Cdk2, Cdk4, CyclinD1, TGF-β, IL-1β, IL-18, Caspase-1, Gapdh* (CST)*, TNF-α, Caspase-1,* and *Gsdmd* antibodies (Abcam).

### CCK8 assay

GC2 cells were treated with different concentrations of MgH_2_. Then, 100 μL of CCK8 working solution was added to each sample. After 1 h of incubation, the OD value at 450 nm was determined by a microplate reader (OA4209, Molecular Devices, China).

### Hydrogen concentration assay

MgH_2_ with a concentration of 10 μg/ml was prepared with deionized water. Hydrogen microelectrode was inserted immediately to detect hydrogen generation. The data was recorded in the subsequent 20 minutes. The hydrogen distribution in the testes of mice was also measured by Hydrogen microelectrode. 0.1 ml of MgH_2_ at 10 μg/ml was administrated with mice by gavage. Anesthesia was performed with pentobarbital sodium and supine position was taken. The testes of mice were exposed by incision along the midline of the abdomen under sterile conditions, and hydrogen microelectrode was inserted into the testes to a depth of about 300 μm for detection. The data was recorded in the subsequent 4 hours.

### Statistical analysis

The data were analyzed by GraphPad Prism 8 (GraphPad software, San Diego, Calif., US) and are expressed as the mean ± standard error (SEM). One-way ANOVA or two-way ANOVA was used to analyze the data. All experiments were repeated at least three times. *P* < 0.05 indicated statistical significance.

## Results

### Characterization and safety evaluation of MgH_2_

**Fig. 1:**
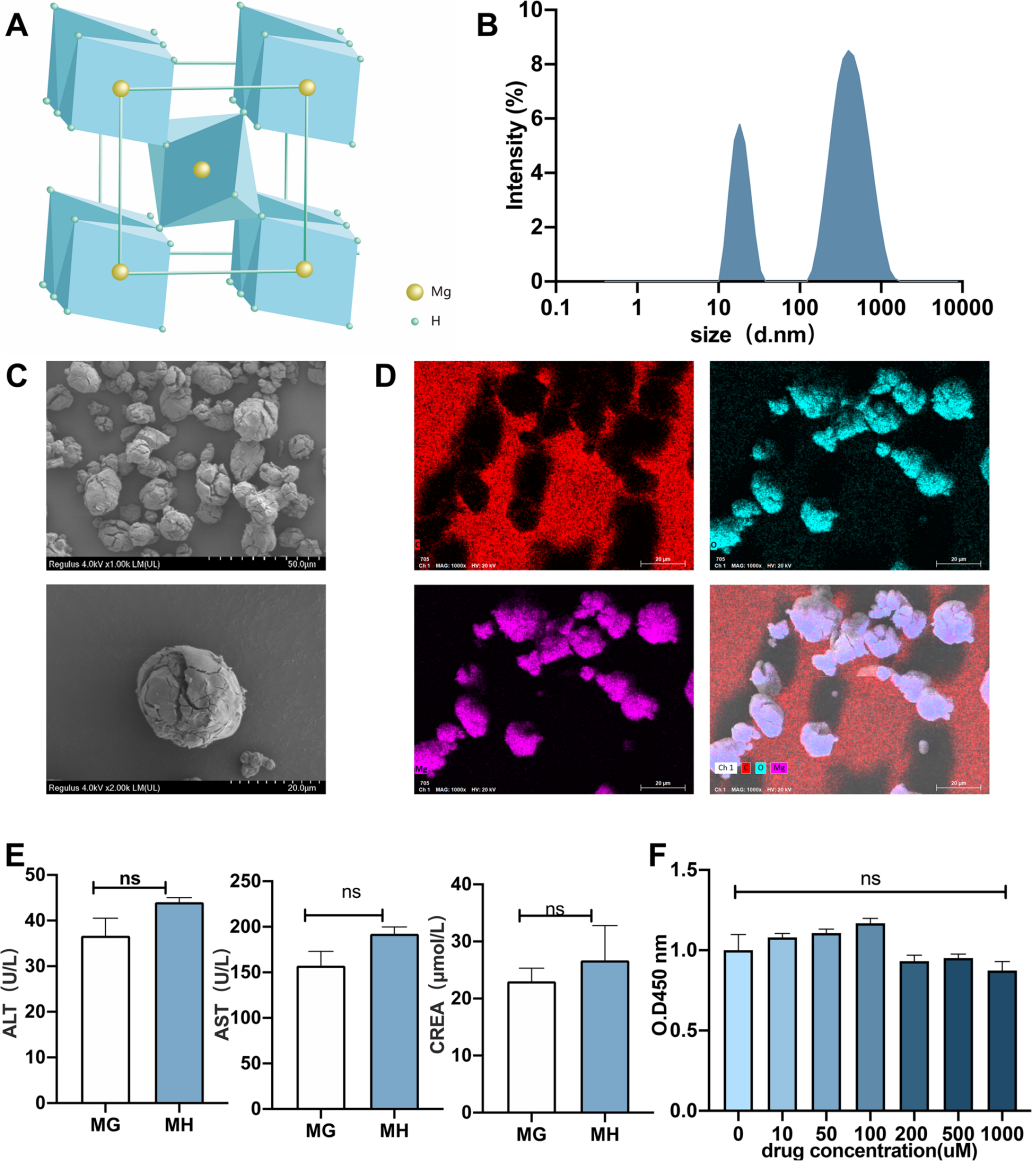
Characterization and safety evaluation of MgH_2_. The MgH_2_ molecular stacking pattern is shown in (**A**). Zetasizer Nano was used to analyze the particle size distribution (**B**). The hydrogen microelectrode was performed to detect the hydrogen generation of MgH_2_ in deionized water (**C**). SEM was used to determine the morphological appearance (**D**) and elemental composition distribution (**E**). The safety of MgH_2_ was also evaluated. Mice were fed MgH_2_-supplemented feed for a long time. On the 29th day, toxicity tests on mouse serum to evaluate liver and kidney function were carried out in MG and MH groups (**F**). In GC2 cells, the cell toxicity (**G**) of MgH_2_ was evaluated by measuring the absorbance at 450 nm after incubation with different concentrations of MgH_2_. The data are expressed as the mean ± SEM, *p < 0.05, **p < 0.01

The MgH_2_ molecular stacking pattern was shown in the Fig. 1A. SEM showed that MgH_2_ was composed of irregular particles with excellent dispersion properties, did not aggregate, and had a uniform particle size distribution, mainly in the range of 100–1000 nm with the main peak at 463.2 nm (Fig. 1B). Notably, there was a secondary peak at 19.02 nm, which indicated the existence of the Mg metal element. When MgH_2_ is scattered to aqueous phase, hydrogen is rapidly generated within 10 min. The hydrogen generation rate started to slow down within 10–25 min, a second peak began to appear after 25 min, and decreased after 30 min (Fig. 1C). SEM also showed that the single MgH_2_ particles had irregular spherical morphologies, with cracking on their surfaces (Fig. 1D). We also analyzed the elemental composition of MgH_2_, and the proportion of Mg was approximately 71.46% (Fig. 1E). The biological toxicity analysis showed that there was no change in the levels of aspartate aminotransferase (AST), alanine aminotransferase (ALT) or serum creatinine (CREM) in MH mice compared with MG mice (Fig. 1F). When the concentration of MgH_2_ was less than 1000 μM, cell viability decreased with increasing drug dose, but the decrease in cell viability did not reach statistical significance (Fig. 1G). In addition, we observed the toxicity of MgH_2_ to GC2 cells after prolonged stimulation (11 days), and the results showed that MgH_2_ had a pro-proliferative effect on GC2 cells, which indicated they were not cytotoxic (Fig. S1D). The results described above indicated that MgH_2_ was safe and nontoxic.

### MgH_2_ protected the reproductive system from radiation-induced injury

**Fig. 2:**
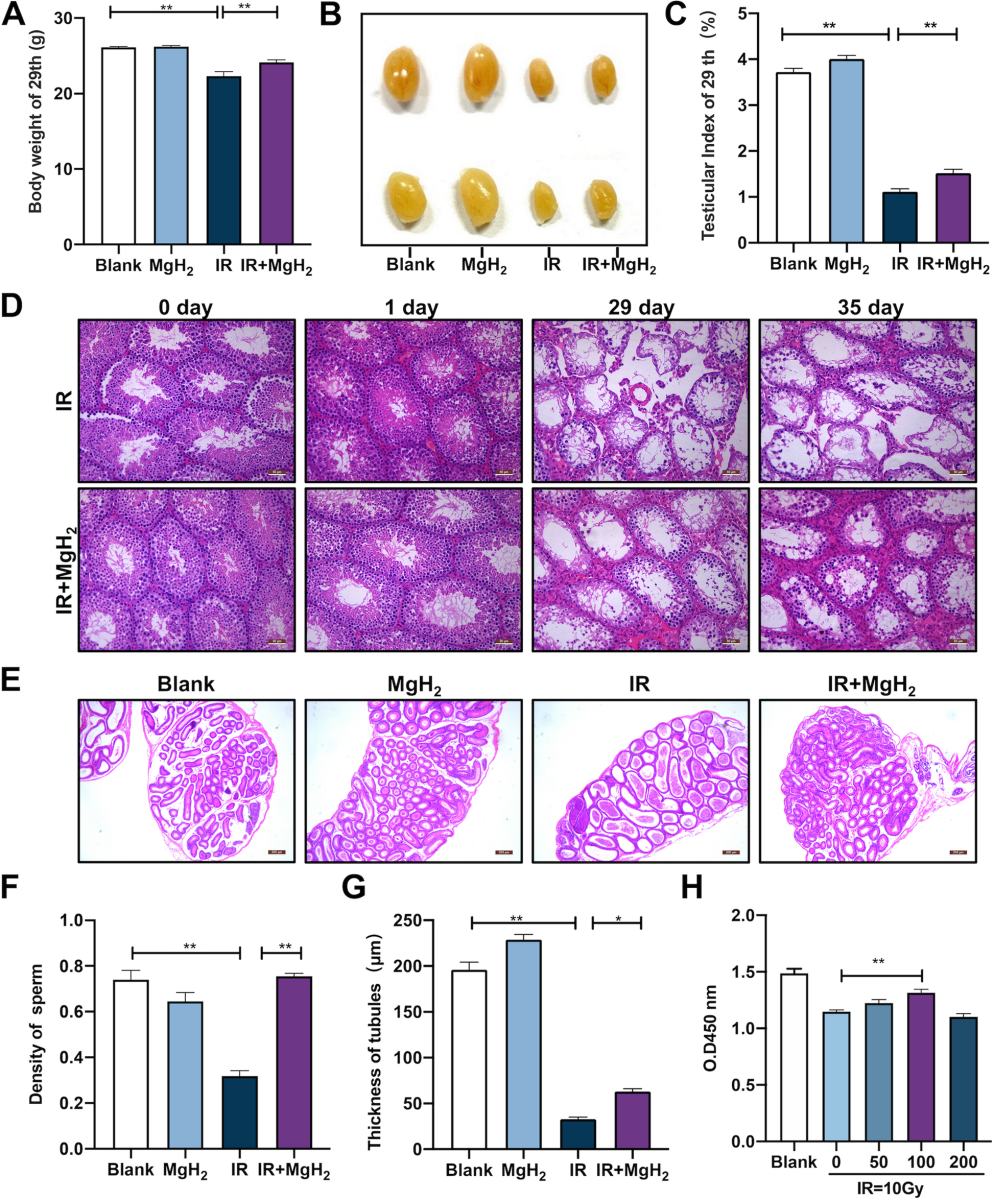
MgH_2_ alleviated irradiation-induced reproductive system damage. The changes in body weight (**A**) and the appearance (**B**) and index (**C**) of the mouse testes were assessed on the 29th day after exposure to 5 Gy whole body irradiation. Then, H&E staining was performed at different time points (0, 1, 29, 35 d) after 5 Gy irradiation of the testes of mice (**D**). Additionally, the epididymis was stained with H&E (**E**) on the 29th day, and the sperm density in each epididymal tubule was measured (*n* = 30) (**F**). 35 day after irradiation, the thickness of each seminiferous tubule was measured (*n* = 20) (**G**). The CCK-8 assay was used to monitor the viability of GC2 (H) cells exposed to 10 Gy irradiation and different concentrations of MgH_2_. Viability was measured at 450 nm. The data are expressed as the mean ± SEM, *p < 0.05, **p < 0.01

Compared with those in the blank group, the body weight (Fig. 2A), testicular size (Fig. 2B) and testicular index (Fig. 2C) of the mice in the IR group decreased significantly 29 d after irradiation, while MgH_2_ treatment significantly ameliorated the IR-induced decreases in body weight, testicular size and testicular index. In the IR-treated mice, H&E staining of the testes revealed pathological damage, including darker eosin staining on the 1st day after IR treatment (Fig. 2D), increased vacuolization, aggravated atrophy, decreased seminiferous tubule thickness and diameter, increased interstitial space and cell number, and increased vacuolization (Fig. 2D) and decreased mature sperm counts in the seminiferous tubules of the epididymis on the 29th day (Fig. 2E, F); additionally, vacuolization of the seminiferous tubules, loose connections of the seminiferous tubules and poor morphology of the interstitial cells were still observed on the 35th day after IR exposure. Moreover, irradiation reduced the thickness of spermatogenic tubules, and MgH_2_ ameliorated this damage (Fig. 2G). However, after MgH_2_ treatment, the IR-induced pathological changes were attenuated. In vitro, 10 Gy irradiation significantly inhibited the viability of GC2 cells (Fig. 2H). However, MgH_2_ treatment at doses ranging from 0 μM to 100 μM ameliorated the IR-induced decrease in cell viability in a dose-dependent manner.

### MgH_2_ improved the decrease in sperm motility and semen quality caused by irradiation

**Fig. 3:**
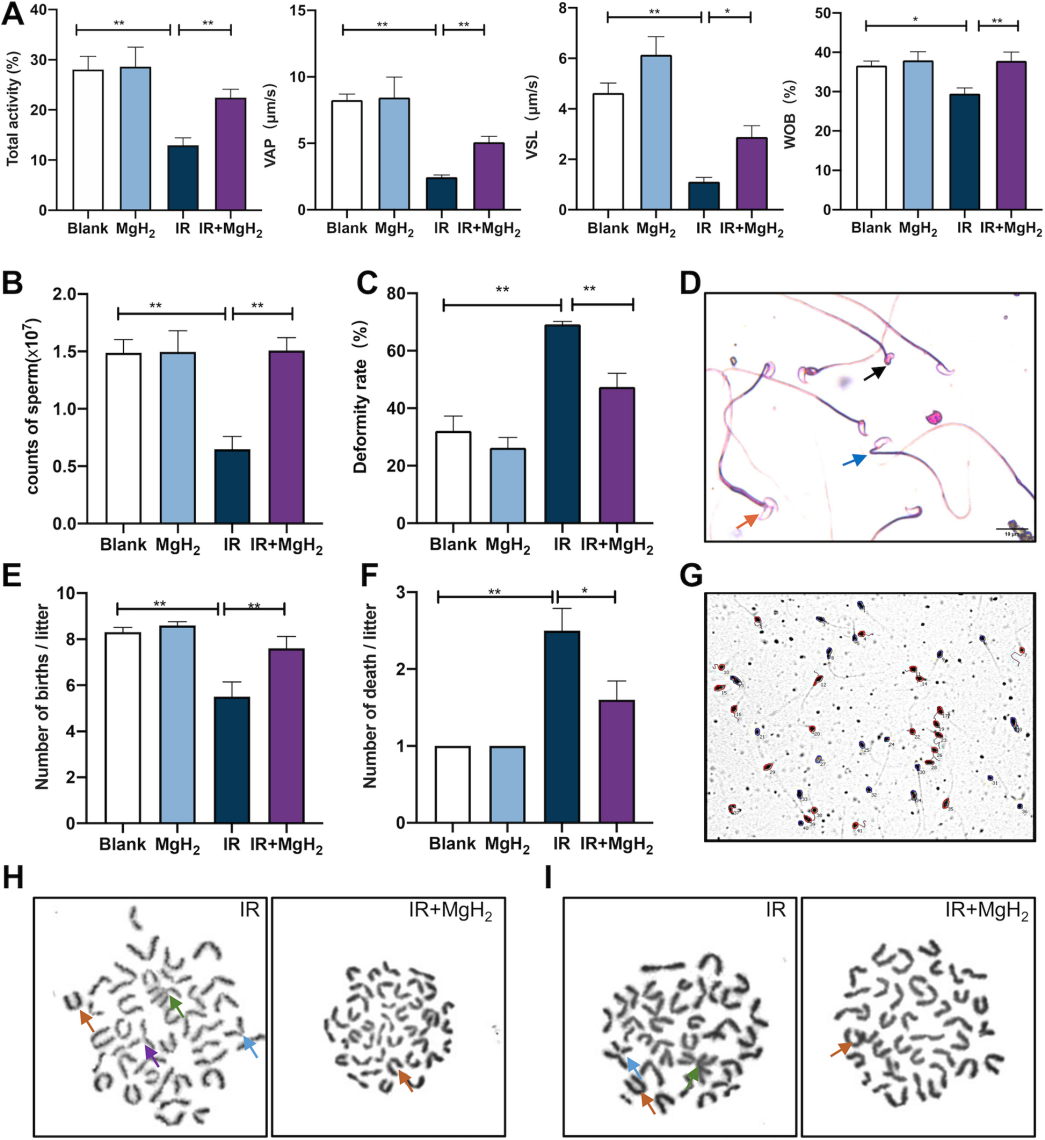
MgH_2_ improved the decrease in sperm motility and quality induced by ionizing radiation. Changes in sperm count and motility were evaluated on the 29th day after 5 Gy whole body irradiation. CASA was used to detect the motility of the sperm from murine epididymis tissues (**A**). The counts of sperm (**B**) and morphological characteristics (**D**) of mouse sperm were observed under a microscope, and the deformity rate (**C**) was assessed (*n* = 200). Then, the genetic effect of MgH_2_ was evaluated in IR-treated mice. After 5 Gy whole body irradiation for 21 days, the number of pups (**E**) and the number of deaths (**F**) per litter were recorded. In addition, changes in the chromosomes of testes in F0 generation mice (**H**) and bone marrow in F1 generation mice (**I**) were also observed under as use Chromosome image analysis system observed under Metafer Automated Slide Scanning Platform. Moreover, the human sperm movement was captured in the diagram (**G**). The data are expressed as the mean ± SEM, *p < 0.05, **p < 0.01

Sperm motility is one of the most important criteria for evaluating sperm quality. Exposure to radiation significantly decreased sperm motility, as shown by decreased VAP, VSL, WOB, total sperm activity (Fig. 3A, Fig. S2) and counts of sperm (Fig. 3B), while MgH_2_ treatment alleviated these changes. Moreover, an increase in deformities, including head deformities, such as banana type (blue) and double head type (blank), and tail deformities, such as tail folding type (orange), were observed in the IR group (Fig. 3C), while MgH_2_ treatment ameliorated these changes (Fig. 3D). Furthermore, experiments investigating genetic effects indicated that IR impaired the fertility of mice, as shown by a decreased litter size per fetus and increased stillbirth rate per fetus in the IR group compared to the blank group (Fig. 3E, F). However, IR-induced changes in fertility were significantly alleviated by MgH_2_ treatment. After irradiation, chromosomal abnormalities were observed in irradiated mice in the F0 generation (Fig. 3H) and nonirradiated mice in the F1 generation (Fig. 3I); these chromosomal abnormalities included fragments (brown) and gaps (blue), and dicentrics (purple) and robertsonian translocations (green). However, MgH_2_ treatment reduced incidence of chromosomal abnormalities. Compared with MG group, the testicular size (Fig. S3A) sperm deformity rate (Fig. S3B), sperm quantity (Fig. S3C), body weight (Fig. S3D) and testicular index (Fig. S3E) of MH group mice were improved. Moreover, the indices of sperm motility in the MH group were consistent with, even better than, those in the MgH_2_ gavage group. Finally, Human sperm trajectories were detected (Fig. 3G), we assessed the protective effect of MgH_2_ on the motility of human sperm exposed to irradiation at a dose of 10 Gy. Compared with IR exposure alone, MgH_2_ treatment significantly improved human sperm motility including total sperm activity, MAD and the sperm counts of type A and B (Table.1).

**Table 1:** Analysis of human sperm motility with or without MgH_2_ treatment after irradiation

Items	Blank	MgH_**2**_	IR	IR + MgH_**2**_
**Total activity (%)**	65.45 ± 11.78 **	68.57 ± 14.35	43.17 ± 10.19	60.14 ± 11.70 **
**A + B (%)**	45.63 ± 13.74 *	49.70 ± 16.27	25.76 ± 10.77	41.76 ± 11.52 **
**MAD (**^**。**^**)**	15.89 ± 2.57 **	16.52 ± 10.59	11.40 ± 2.26	15.28 ± 3.37 **
**VCL (μm/s)**	40.14 ± 10.09	43.27 ± 10.59	34.59 ± 9.52	38.97 ± 8.69
**VSL (μm/s)**	22.05 ± 5.10	25.36 ± 8.08	18.46 ± 8.98	21.79 ± 6.88
**VAP (μm/s)**	36.72 ± 9.27	39.69 ± 9.98	31.51 ± 9.25	35.52 ± 8.27
**ALH (μm)**	4.03 ± 0.04	4.05 ± 0.07	3.99 ± 0.09	4.02 ± 0.07
**LIN (%)**	55.32 ± 4.08	57.60 ± 8.16	50.57 ± 10.46	54.56 ± 7.43
**WOB (%)**	90.15 ± 2.76	91.52 ± 1.63	90.11 ± 2.09	90.91 ± 1.49
**SRT (%)**	60.71 ± 3.95	62.80 ± 7.78	55.88 ± 10.26	60.71 ± 7.72

*n* = 11 for each group, values are expressed as Mean ± SD, * *P* < 0.05, **P < 0.01 vs IR group

### MgH_2_ maintained spermatogenesis in male mice after irradiation exposure

**Fig. 4:**
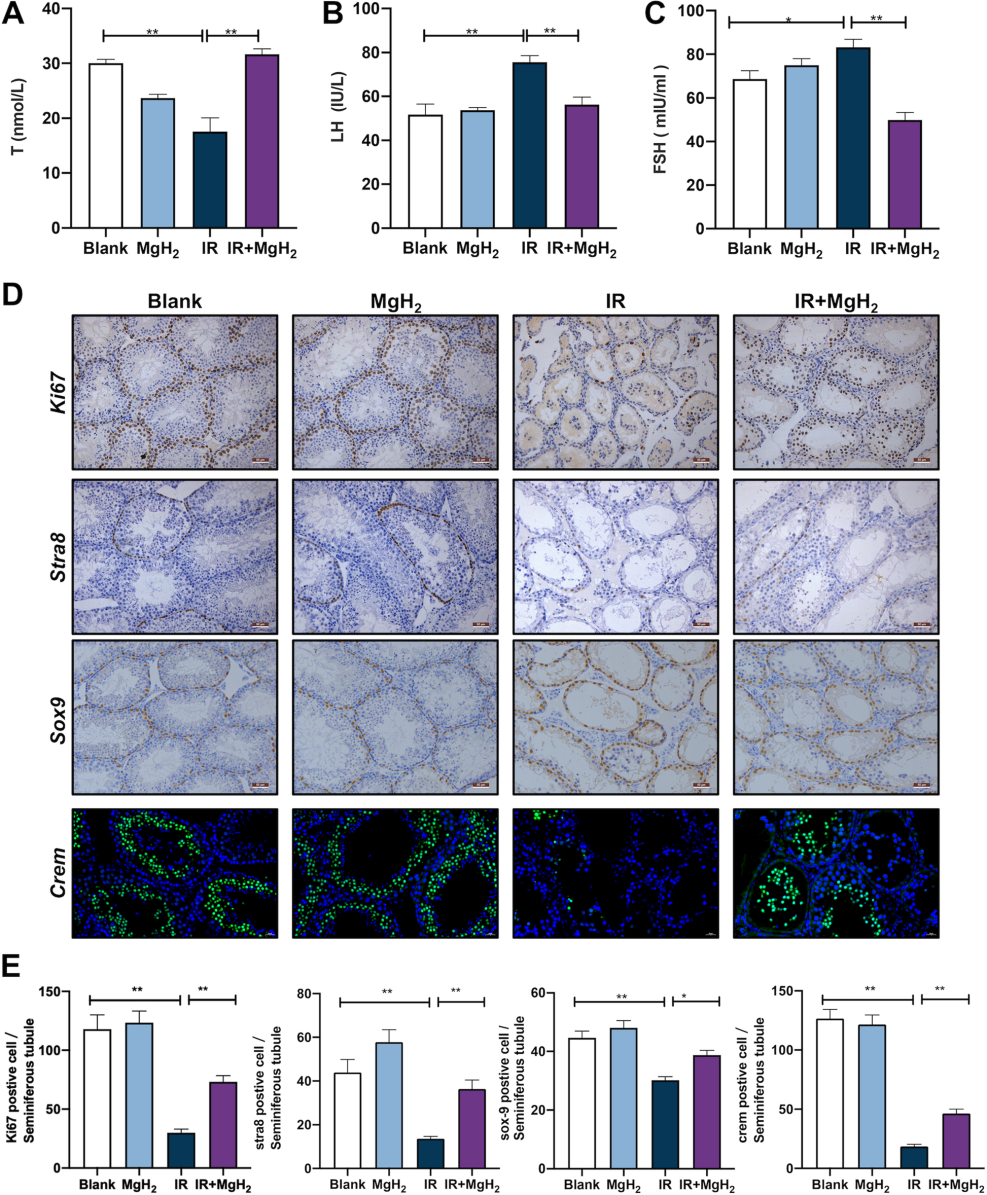
MgH_2_ maintained sperm development in male mice after exposure to ionizing radiation. The T (**A**), FSH (**B**) and LH (**C**) levels in the serum 12 hours after irradiation were also detected. Moreover, Changes in *Ki67, Stra8, Sox-9* and *Crem* (**D**) expression in mouse testes were detected by IHC staining on the 29th day after 5 Gy whole body irradiation, and the positive rates were analyzed and counted (**E**). The data are expressed as the mean ± SEM, * *p* < 0.05, ** *p* < 0.01

After irradiation exposure, a significantly decreased serum T levels (Fig. 4A) and increased serum LH and FSH levels were observed (Fig. 4B, C). Subsequently, spermatogenesis and development were assessed. The positive rates of spermatogonia (*Stra8*), Sertoli cells (*Sox-9*) and round sperm cells (*Crem*) were significantly decreased in the testes after irradiation exposure, but the numbers of these cells were rescued by MgH_2_ treatment (Fig. 4D, E). The results indicated that MgH_2_ could promote the recovery of spermatogenic function after ionizing radiation exposure, mainly by promoting hormone secretion and maintaining spermatogonia, Sertoli cell and round sperm cell populations.

### MgH_2_ suppressed apoptosis, pyroptosis and inflammation and alleviated cell cycle arrest by regulating ROS in IR-induced male infertility

**Fig. 5:**
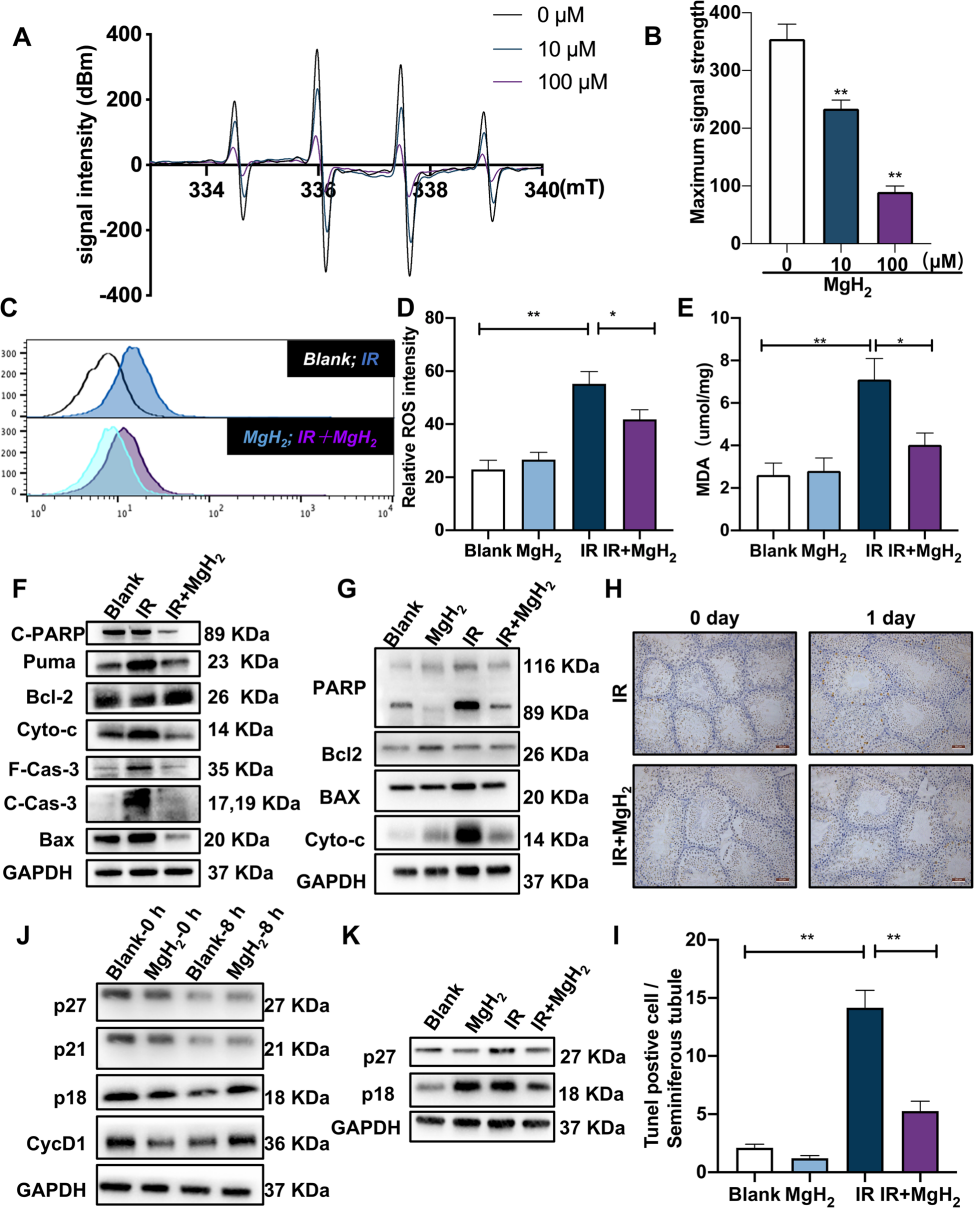
MgH_2_ regulated inflammation, apoptosis, pyroptosis and cell cycle progression by suppressing oxidative stress. ESR assays were used to measure the ability of different concentrations of MgH_2_ to scavenge the [·OH] generated by the Fenton reaction (**A**), and the peak value of the ESR spectrum was recorded (**B**). The hydrogen microelectrode was performed to monitor the hydrogen distribution in the testes of mice(**C**). ROS generation in GC2 cells irradiated with 10 Gy was detected by flow cytometry(**D**), and the positive rates were recorded (**E**). Subsequently, the levels MDA (**F**) in the testes 12 hours after 5 Gy irradiation were detected, and the effect of MgH_2_ on the level of apoptosis at 12 h after IR was evaluated. The expression of apoptosis-related proteins in testes (**G**) and GC2 cells (**H**) was detected. The testis tissue was stained for *Tunel* assay (**I**), and the number of *Tunel*-positive cells in each seminiferous tubule was counted (**J**). The effect of MgH_2_ on cell cycle-related protein expression in GC2 cells was also detected after MgH_2_ administration alone (**K**) and 48 h (**L**) after irradiation. The data are expressed as the mean ± SEM, * *p* < 0.05, ** *p* < 0.01

To detect the [·OH] scavenging ability of MgH_2_, four characteristic ESR spectra with an intensity ratio of 1:2:2:1 were recorded (Fig. 5A). [·OH] levels gradually decreased with increasing MgH_2_ concentration, which confirmed the ability of MgH_2_ to scavenge free radicals (Fig. 5B). The hydrogen microelectrode was performed to analyze the distribution of H_2_ in mouse testis after gavage of MgH_2_. The results showed that the generated H_2_ from MgH_2_ had been detected 1 hour after administration in the testis, and been increasing in the subsequent period, which indicated that MgH_2_ can indeed release H_2_ in mice and can distribute to the testis. (Fig. 5C). Furthermore, MgH_2_ treatment obviously reversed the IR-mediated increase in ROS levels in GC2 cells (Fig. 5D, E). In addition, irradiation exposure resulted increased MDA contents in the testes, while MgH_2_ treatment reversed these effects (Fig. 5F).

Apoptotic protein expression was further investigated. In testicular tissue, IR significantly increased the expression of *Bax*, *Puma*, *Cyto-c* and *Caspase-3*. Interestingly, MgH_2_ administration significantly downregulated the expression of these proteins (Fig. 5H). Similar results were also observed in GC2 cells (Fig. 5H). In addition, *Tunel* staining confirmed that MgH_2_ inhibited IR-induced apoptosis in the testes (Fig. 5I, J). Moreover, increased ROS levels also contribute to IR-induced cell cycle arrest. The expression of *p21* and *p27* in GC2 cells was increased 8 hours after treatment with MgH_2_ alone (Fig. 5K), which indicated that the cells were arrested in the G0/G1 phase by MgH_2_. However, 48 h after irradiation, the expression of *p21* and *p27* was decreased while the expression of *Cdk2* and *CyclinD1* was increased in GC2 cells (Fig. 5L), which indicated that MgH_2_ could cause irradiated spermatogonia to quickly progress past the GO/G1 phase, effectively alleviating the G2/M phase cell cycle arrest induced by irradiation. IR-induced ROS production can lead to inflammatory reactions through the pyroptosis pathway. The increased expression of pyroptosis-related proteins, including *Gsdmd*, *Caspase-1*, *IL-1β* and *IL-18*, was significantly increased after ionizing radiation exposure, and this effect was reversed after MgH_2_ treatment. In addition, MgH_2_ treatment significantly ameliorated the irradiation-induced increase in *TGF-β* and *TNF-α* expression, thus suppressing inflammation (Fig. S4).

## Discussion

Ionizing radiation has been generally applied in disease diagnosis and cancer treatment, but IR-induced damage to tissues that are adjacent to carcinoma tissues is inevitable. Testicular sensitivity to radiation makes this tissue more prone to damage during radiotherapy, leading to temporary or permanent gonadal toxicity in males [13]. Irradiation-induced ROS production has been proven to be the key process in the development of IR-induced testis damage [21]. However, few ROS-targeting agents have progressed to clinical trials for the treatment of IR-induced testis injury due to poor antioxidant capacity. Therefore, the development of novel therapeutic agents remains necessary. In this study, we revealed the structure and biosafety of MgH_2_; confirmed the potential radioprotective effect of MgH_2_ in improving sperm motility, semen quality and spermatogenesis, and thus male fertility, after ionizing radiation exposure; and revealed the underlying mechanisms, including the suppression of the inflammation, apoptosis, pyroptosis and cell cycle arrest through inhibiting the oxidative stress induced by IR.

Nanobiology broadens strategies for disease diagnosis and treatment, and it can improve the effect of radiotherapy [16]. To avoid the shortcomings of the radiotherapeutic approaches currently used for cancer treatment, it is necessary to protect healthy tissues from irradiation-induced damage, and more effective treatments must be developed to reduce the harmful side effects caused by irradiation [17]. The most important feature of nanomaterials is their increased specific surface area, which means that a material in the form of a nanoparticle will be more active than in the form of a larger particle with the same mass [17]. We prepared MgH_2_ in the form of a powdery solid, which could release H_2_, and this preparation has not previously been reported in the biomedical field or used the context of ionizing radiation. MgH_2_ is stable and has a hydrogen storage capacity of 7.6 wt% [18]. The storage of hydrogen in the form of MgH_2_ is a promising technology that currently being used in the development of new energy vehicles and fuel cells and has achieved safer hydrogen transportation [22]. In this study, the size of MgH_2_ micro/nanoparticles was mainly distributed in the range of 100–1000 nm, SEM showed that single MgH_2_ particles had an irregular spherical morphology and a particle size of approximately 463.2 nm (Fig. 1B), and Mg accounted for approximately 71.46% of the particles (Fig. 1E). Radiation can arrest cell proliferation, deplete spermatogonia, and lead to testicular weight loss. Body weight and organ index are important parameters for evaluating the toxicity and efficacy of drugs in mammals [23]. MgH_2_ is safe and nontoxic. The body weight and testicular index of mice exposed to IR decreased significantly, while MgH_2_ treatment ameliorated this effect (Fig. 2 A-C). On the other hand, we added MgH_2_ into feed, in which the total dose of MgH_2_ reached 150 mg/kg. 29 days after feeding, the indices related to liver and renal function were not changed, which indicated that MgH_2_ was indeed safe and nontoxic (Fig. 1F). In addition, there were no significant differences in body weight, food intake, water intake and total food intake between MH group and MG group (Fig. S1). These results suggest that MgH_2_ is safe and does not have long-term toxicity.

At present, H_2_ has been proven to be a safe and effective agent for protecting against radiation [14]. H_2_ has many advantages in that it can effectively neutralize [·OH] in living cells; penetrate biofilms; diffuse to the cytoplasm, mitochondria and nucleus; and reduce cytotoxic free radicals [13]; thus, H_2_ protects against radiation in vitro and in vivo. Treatment with H_2_ before irradiation could significantly suppress the apoptosis induced by ionizing radiation and increase cell viability [15]. MgH_2_, an excellent hydrogen storage material, released a large amount of H_2_ into the aqueous phase, and MgH_2_ exhibited inherent promise of becoming a radioprotective agent. Furthermore, MgH_2_ can indeed release H2 in mice and can distribute to the testis. (Fig. 5C)*.*

The indirect effect of dissociated water produced after IR exposure can lead to testis injury [24]. Oxidative stress is the result of an imbalance between the generation and consumption of excessive ROS and the decreased activity of the antioxidant defense system [25]. The generation of ROS impairs male reproductive function and leads to infertility. ROS are a broad class of molecules, among which [·OH] is the most active product in cells and easily attacks cellular macromolecules, such as DNA, protein and lipids, exerting strong cytotoxic effects [26]. Sixty to 70 % of IR-induced tissue damage is caused by [·OH] [12]. IR can generate ROS through the IR-induced decomposition, excitation and decay of water molecules [27]. IR can also directly lead to increased mitochondrial ROS production. Radiation-induced ROS overproduction can be amplified by a mechanism involving mitochondrial permeability transitions. At present, an increasing number of free radical scavengers have been identified. MgH_2_ can quickly produce hydrogen in water (Fig. 1C), and hydrogen molecules have the ability to scavenge free radicals. In addition, hydrogen has the lowest relative molecular weight and can freely penetrate into mitochondria, which can protect them from oxidative damage. Therefore, we believe that MgH_2_ inhibits ROS production by removing IR-induced [·OH] by generation of hydrogen. In addition, the contents of malondialdehyde (MDA) is one of the indices used to evaluate oxidative damage [28]. In this study, MgH_2_ treatment significantly reversed the increase in MDA contents observed after irradiation. Therefore, MgH_2_ can also act as an exogenous antioxidant to protect male fertility by effectively scavenging free radicals (Fig. 5F).

At present, ROS are considered an important cause of testicular dysfunction, as shown by the decrease in sperm motility and semen quality [29]. Sperm malformation is closely related to sperm function [26]. It was reported that among 134 health care workers exposed to ionizing radiation, abnormal sperm morphology and decreased sperm vitality were significantly increased [30]. In our study, sperm generation notably increased after MgH_2_ treatment, and the motility and deformity rate were markedly improved (Fig. 3A-D). On the other hand, the sperm motility results in MH mice were consistent with those in mice treated with MgH_2_ via intragastric administration (Fig. S2). Additionally, the effect of MH group was stronger. MgH_2_ could not only improve sperm motility in mice but also promote semen quality in humans (Table 1). Sperm abnormalities significantly affect the development of offspring. MgH_2_ could protect fertility by improving sperm motility and quality, and MgH_2_ has the potential to be applied as a food additive or health care product.

A large amount of ROS can lead to meiosis inhibition, chromosome abnormalities and spindle microtubule deformation [31]. Paternal exposure to irradiation not only increases the rate of germ cell mutation in F0 male mice directly exposed to irradiation but also may lead to genetic instability in the unexposed F1 generation. Paternal exposure to irradiation significantly increases the rate of chromosome abnormalities observed in the F1 generation [32]. In our study, MgH_2_ effectively removed excessive ROS levels in cells and maintained mitochondrial activity; thus, MgH_2_ protected the normal morphology of spindles and chromosomes in F0 generation testes (Fig. 3H) and F1 generation bone marrow (Fig. 3I). Additionally, we found that MgH_2_ could increase the number of offspring born in each litter, reduce the number of stillbirths, and ameliorate the IR-induced genetic aberration observed the F1 generation (Fig. 3E, F).

Spermatogenesis consists of three consecutive phases: the mitosis phase (proliferation and differentiation of spermatogonia), the meiosis phase (generation of haploid sperm cells via meiosis of spermatocytes) and the spermatogenesis phase (elongation and maturation of sperm cells) [33]. This process is regulated by various hormones. Therefore, damage during any phase affects spermatogenesis. The spermatogenic cycle of male mice last approximately 35 days, at which time spermatogonia begin to recover and new spermatogenic cells appear [34]. In our study, the structure of the seminiferous tubules in IR-treated mice, including the widened interstitial space, the decreased number of interstitial cells, and the serious vacuolation, had not recovered on the 35th day. After MgH_2_ treatment, the structure of the testis and seminiferous tubules was effectively improved, and new spermatogonia could be observed (Fig. 2D). In addition, atrophy of the germinal epithelium of the seminiferous tubules and a decrease in spermatogenic cell counts can manifest as a decrease in seminiferous tubule thickness. MgH_2_ improved the reduction in seminiferous tubule thickness induced by IR (Fig. 2G). T is a necessary prerequisite for spermatogenesis and plays an important role in male fertility and spermatogenesis, and T also maintains the structure and normal physiological function of seminiferous tubules [23, 35]. IR destroys the function of testicular interstitial cells via ROS generation, resulting in a decrease in T secretion, which eventually leads to disrupted spermatogenesis, sperm reduction and testicular atrophy. T is mainly secreted by interstitial cells, which originate in the hypothalamus and secrete gonadotropin-releasing hormone (GnRH). In turn, GnRH stimulates the pituitary gland to generate LH and FSH. In an early report, the testis had already sustained interstitial cell damage after exposure to radiation at a dose of approximately 200 cGy [36]. Additionally, there is a risk of permanent and persistent deficiency in T levels with the increase in LH levels, which may eventually lead to hypogonadism. MgH_2_ treatment restored the T, LH and FSH levels to their normal control levels (Fig. 4A-C).

In the process of spermatogenesis, Sertoli cells (SCs) provide structural support for sperm cells, determine the volume of the testes and seminiferous tubules, and produce a series of proteins involved in the proliferation, differentiation and metabolism of germ cells [37]. Active spermatogonia are most vulnerable to IR-induced germ cell apoptosis, while SCs are less sensitive; dosages as low as 0.1 Gy can damage spermatogonia, while damage to SCs only occurs with a larger dose [38]. In this study, we found that the expression of *sox-9* in SCs, *stra8* in spermatogonia and *crem* in round sperm cells was significantly increased (Fig. 4D-E). These results indicated that MgH_2_ participated in spermatogenesis and protected sperm development in three stages: the generation of Sertoli cells, spermatogonia and round sperm cells.

The irradiation-induced increase in ROS production is also related to the increase in apoptosis [39]. Mitochondria are an important organelle for the process of apoptosis, which is mainly triggered by the release of *Cytochrome C* from the mitochondria into the cytoplasm and is mainly mediated by *Bcl2* family proteins [24]. *Bax*, an apoptosis-promoting factor, is located in the cytosol. However, during apoptosis, *Bax* translocates to mitochondria and releases *Cytochrome C* from the mitochondria to the cytoplasm to induce apoptosis [40]. In addition, *Caspase-3* is a co-effector molecule of many apoptosis pathways, and its cleavage is considered the main terminal cleavage event in the apoptosis process. MgH_2_ treatment inhibited the upregulation of *Bax* expression and the activation of *Caspase-3* (Fig. 5G). In addition, *Tunel* staining showed that the apoptosis rate in seminiferous tubules increased significantly on the 1st day after IR exposure, while MgH_2_ treatment significantly ameliorated the apoptosis induced by IR (Fig. 5I-K). Similarly, MgH_2_ also exerted an antiapoptotic effect in GC-2 cells (Fig. 5H). Some studies have shown that ROS may promote inflammation by activating inflammasomes [41]. The imbalance of proinflammatory factors and anti-inflammatory factors aggravates damage to tissues and organs. *Gsdmd* is a key molecule in the pyroptosis pathway, and it mainly relies on *Caspase-1/11* for cleavage and activation; the cleavage of *Gsdmd* leads to the maturation and release of the downstream inflammatory cytokines *IL-1β* and *IL-18* and triggers inflammatory reactions. MgH_2_ could treatment significantly reduce the expression of *Gsdmd*, *Caspase-1*, *IL-1β* and *IL-18* after irradiation exposure, which indicated that MgH_2_ could also reduce radiation-induced damage and protect fertility by targeting the pyroptosis pathway (Fig. S4).

Dysregulated cell cycle progression is also one of the most important biological effects caused by ionizing radiation, and cell cycle arrest after irradiation exposure is related to the increase in ROS levels in mitochondria. IR can induce cell cycle arrest at the G1/S and G2/M phases, and of these phases, the G2/M phase is the most sensitive to ionizing radiation [42]. The cell cycle progression of irradiated human lung cancer cells can be inhibited by G2 arrest [43]. The ability of IR to cause G1-phase arrest depends on the accumulation of *p53*, which is mainly mediated by *p21/p27*, which belong to the *Cip/Kip* family and have specifically bind to and inhibit most *Cdk/Cyclin* complexes. In addition, the *p21* protein can bind to the *Cyclin-E-Cdk2* complex and inactivate it, resulting in cell cycle arrest at the G1/S phase transition [44]. First, we found that after 8 h of MgH_2_ treatment, *p21* and *p27* expression was significantly increased, causing cells to arrest at the G0/G1 phase before exposure to irradiation. After IR exposure, the expression of *p21/p27* was increased, and MgH_2_ reversed this trend and increased the levels of *Cdk2* and *CyclinD1* so that the cells quickly progressed from the G2/M phase (Fig. 5K, L). These results indicated that MgH_2_ could effectively ameliorate ionizing radiation-induced cell cycle arrest in the G2/M phase.

## Conclusion

In this study, we revealed the structural characteristics and excellent dispersion properties of MgH_2_; verified the biosafety of MgH_2_ in vivo and in vitro; confirmed the potential radioprotective effects of MgH_2_ on male fertility, including the improvement of sperm motility and the maintenance of spermatogenesis after ionizing radiation exposure; and revealed the mechanisms by which MgH_2_ suppresses the inflammation, apoptosis, and pyroptosis and the ameliorates the cell cycle arrest induced by irradiation through mediating oxidative stress.

## Supplementary Information

**Additional file 1: Figure S1.** The changes of food intake (A), water consumption (B) and body weight (C) of mice in MG group and MH group within 29 days after irradiation were recorded. The toxic effect of MgH_2_ on GC2 cells after prolonged stimulation (11 Days)(D). The data are expressed as the mean ± SEM.

**Additional file 2: Figure S2.** The analysis of mouse sperm motility (VCL, ALH, MAD, LIN and SRT) on the 29th day after 5 Gy irradiation. The data are expressed as the mean ± SEM, * *p* < 0.05, ** *p* < 0.01.

**Additional file 3: Figure S3.** Testicular characteristic diagram (A) of MG and MH mice on the 29th day after irradiation, as well as the changes of deformity rate (B), sperm quantity (C), mouse body weight (D), testicular index (E) and sperm motility (F), including total sperm motility, ALH, VSL, VCL, VAP, LIN, MAD, WOB and SRT. The data are expressed as the mean ± SEM, * *p* < 0.05, ** *p* < 0.01.

**Additional file 4: Figure S4.** Pyroptosis and inflammation related proteins in the testis of mice 12 hours after 5 Gy irradiation were analyzed with or without MgH_2_ treatment.

## Data Availability

All data and materials are available.
