# Supplementary figures and images for: The hydrogen storage nanomaterial MgH2 improves irradiation-induced male fertility impairment by suppressing oxidative stress
Source: Biomater Res. 2022 May 26;26:20. doi: 10.1186/s40824-022-00266-6 (PMC9134580; doi:10.1186/s40824-022-00266-6)

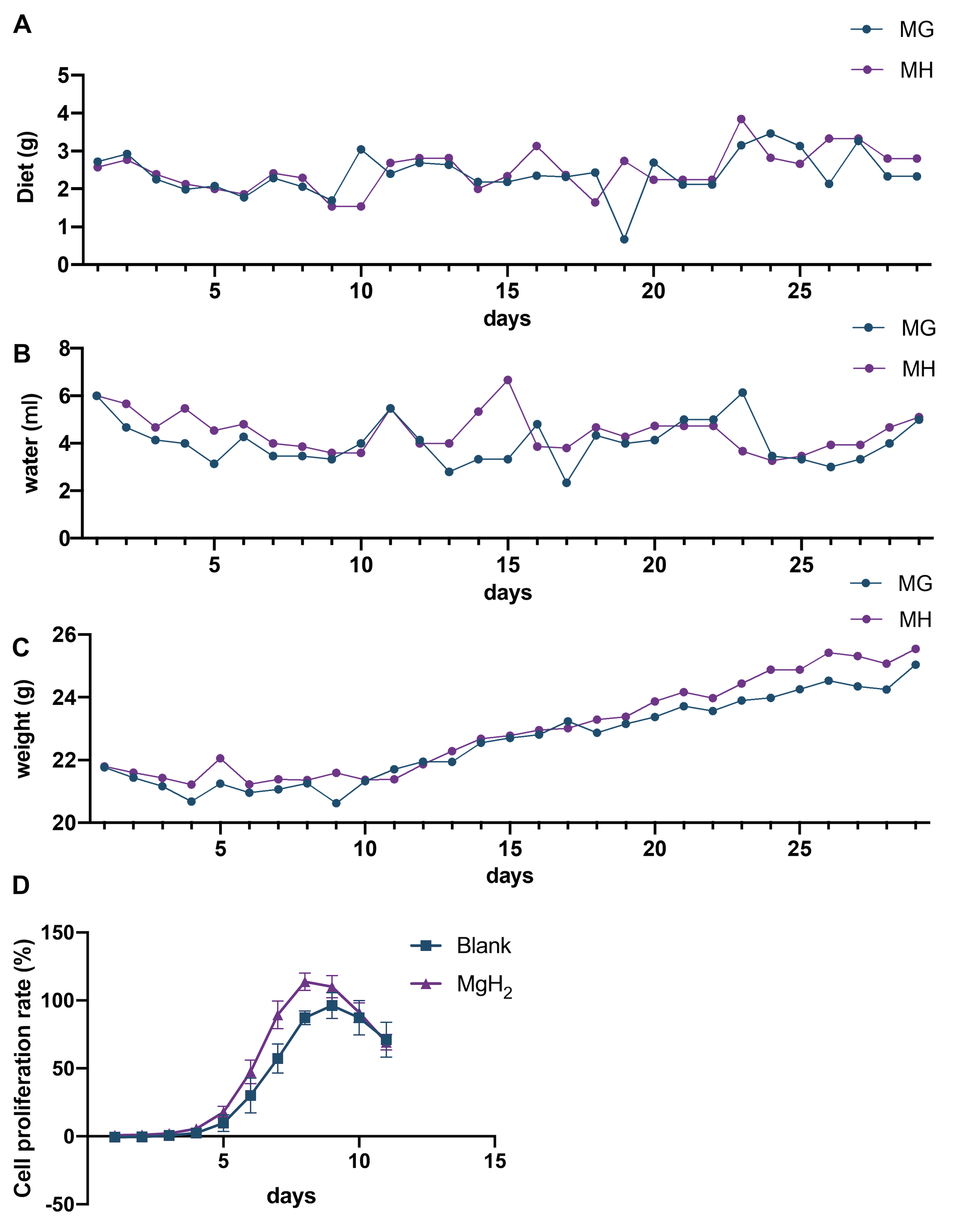

Supplement: Supplementary file 1 — Additional file 1: Figure S1. The changes of food intake (A), water consumption (B) and body weight (C) of mice in MG group and MH group within 29 days after irradiation were recorded. The toxic effect of MgH2 on GC2 cells after prolonged stimulation (11 Days)(D). The data are expressed as the mean ± SEM. [file 40824_2022_266_MOESM1_ESM.tif]

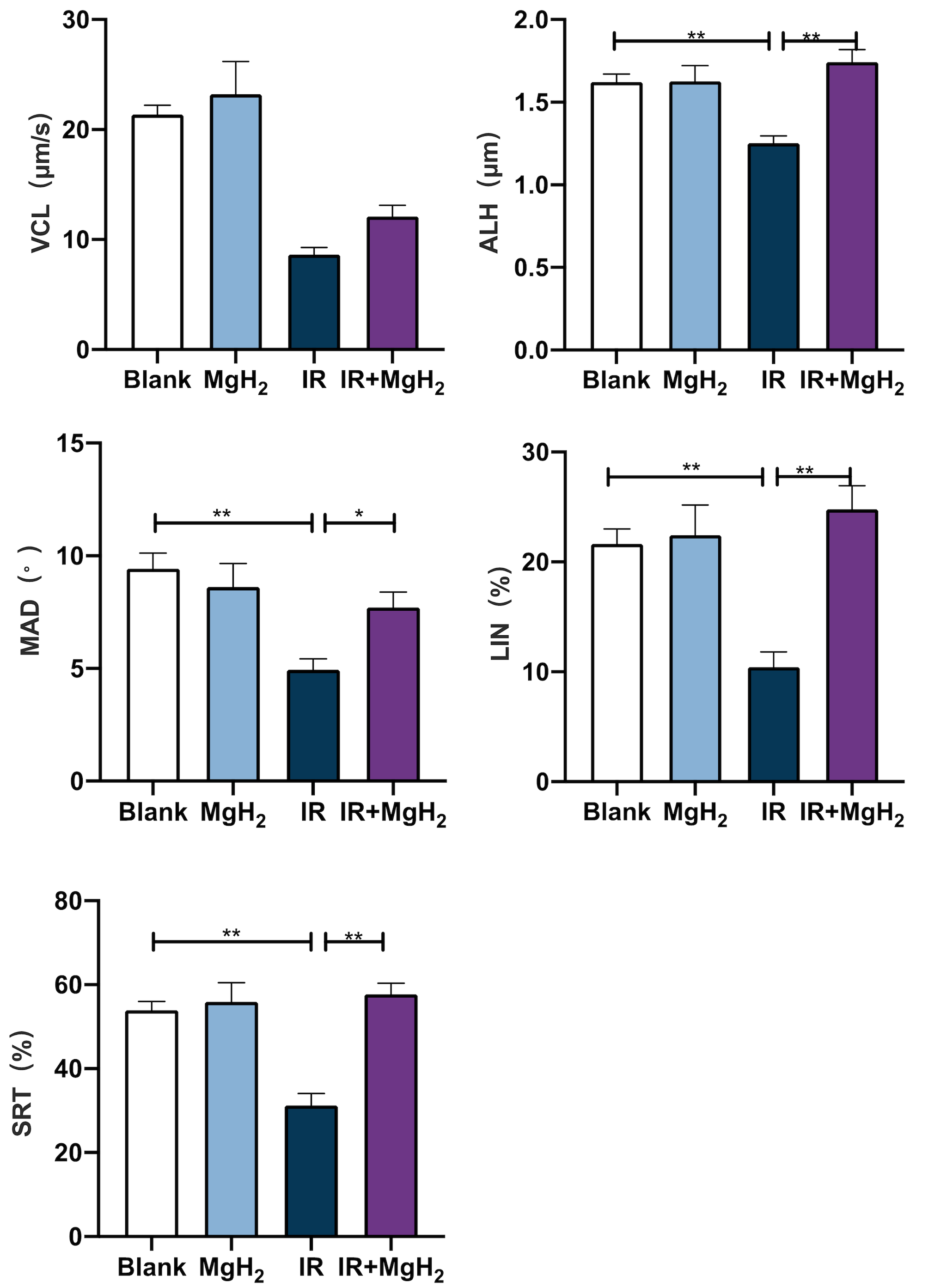

Supplement: Supplementary file 2 — Additional file 2: Figure S2. The analysis of mouse sperm motility (VCL, ALH, MAD, LIN and SRT) on the 29th day after 5 Gy irradiation. The data are expressed as the mean ± SEM, * p < 0.05, ** p < 0.01. [file 40824_2022_266_MOESM2_ESM.tif]

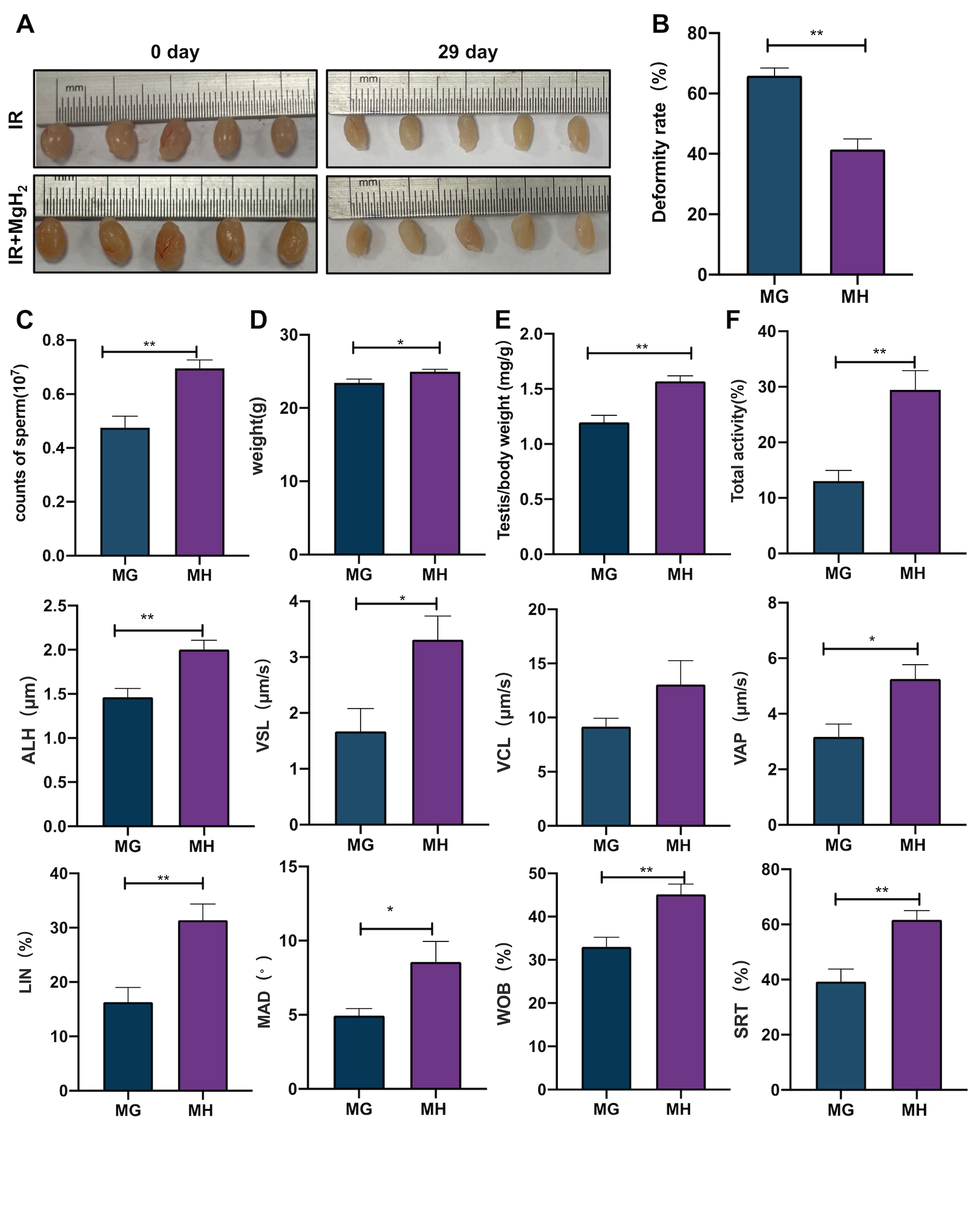

Supplement: Supplementary file 3 — Additional file 3: Figure S3. Testicular characteristic diagram (A) of MG and MH mice on the 29th day after irradiation, as well as the changes of deformity rate (B), sperm quantity (C), mouse body weight (D), testicular index (E) and sperm motility (F), including total sperm motility, ALH, VSL, VCL, VAP, LIN, MAD, WOB and SRT. The data are expressed as the mean ± SEM, * p < 0.05, ** p < 0.01. [file 40824_2022_266_MOESM3_ESM.tif]

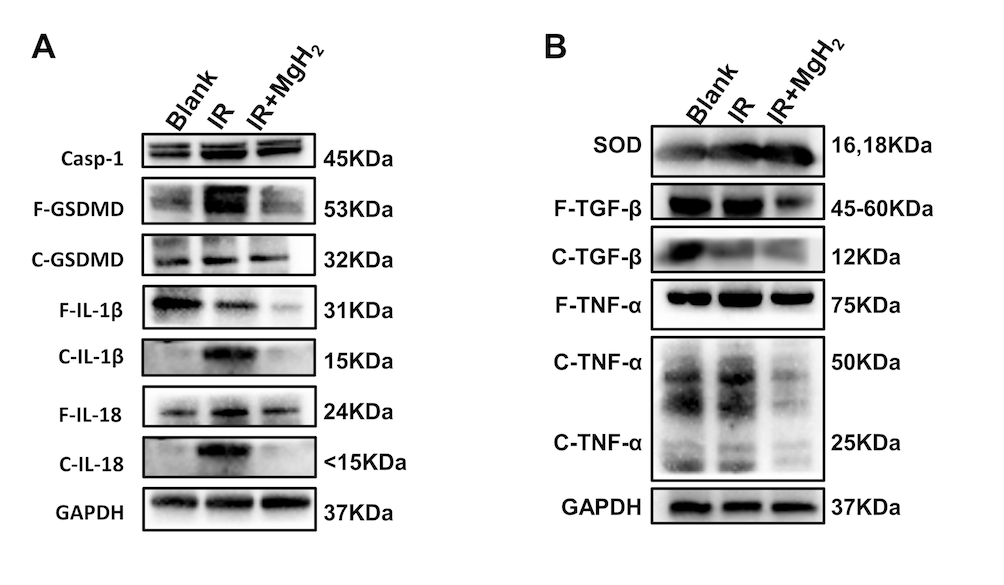

Supplement: Supplementary file 4 — Additional file 4: Figure S4. Pyroptosis and inflammation related proteins in the testis of mice 12 hours after 5 Gy irradiation were analyzed with or without MgH2 treatment. [file 40824_2022_266_MOESM4_ESM.tif]
